# Effect of amino acids lysine and arginine on fracture healing in rabbits

**DOI:** 10.4103/0019-5413.65137

**Published:** 2010

**Authors:** Marcos Almeida Matos

**Affiliations:** *Bahian School of Medicine and Public Health, Salvador-Bahia, Brazil*

Sir

I would like to congratulate the authors of the report “Effect of amino acids lysine and arginine on fracture healing in rabbits: a radiological and histomorphological analysis” for their important and useful findings.^1^ However, I would like the authors to make some points and ideas clearer or give their opinions.

In the section “gross examination and manual palpation” the authors declare that specimens graded 1 and 2 were broken by twisting or loading. It probably means that those broken specimens were histologicaly assessed in the same way as non-broken specimens; I did not understand whether the authors considered one or both sides of the callus in such a case. Additionally, I did not understand if the authors performed transverse or longitudinal cuts for histological examination of the specimens or what type of parameters they have chosen to determine callus area.^2^,^3^

Histomorphometric evaluation using decalcified bone stained with hematoxyline and eosine has been described in several articles,^2^ and I believe it would have been useful in this case. This kind of evaluation could assess the changes which might have taken place in the bone metabolic unit.

Despite the possible minor oversights, I would like to praise the authors for such an intelligent writing, which can be seen in many places of the article. The discussion about the hypothesis that arginine effect on bone healing can be partially explained by nitric oxide production, for instance, demonstrates the authors’ expertise.

## References

[1] Sinha S, Goel SC (2009). Effect of amino acids lysine and arginine on fracture healing in rabbits: A radiological and histomorphological analysis. Indian J Orthop.

[2] Matos MA, Araujo FP, Paixao FB (2008). Histomorphometric evaluation of bone healing in rabbit fibular osteotomy model without fixation. J Orthop Surg Res.

[3] Matos MA, Goncalves RR, Araujo FP (2001). Experimental model for osteotomy in immature rabbit. Acta Ortop Bras.

